# Hypoxia to the Rescue: When Oxygen Therapies Backfire

**DOI:** 10.1371/journal.pbio.0030211

**Published:** 2005-05-03

If a novel oxygen-producing metabolic pathway hadn't evolved in ancient microbes over 3 billion years ago, it's unclear whether humans and other oxygen-dependent species would have either. But evolve we did, adapted to having just the right level of oxygen coursing through our blood—too little oxygen (hypoxia) causes headaches, nausea, and eventually death. Patients with acute respiratory distress syndrome (ARDS) and other serious lung injuries routinely receive oxygenation therapy to facilitate oxygen delivery to deprived tissues. But too much oxygen (hyperoxia) kills, too. Hyperoxia produces free radicals, causing oxidative damage to cells and tissues by disrupting cellular components. And recent evidence suggests that oxygenation therapy might produce dangerous side effects in patients with ARDS who also have severe pulmonary inflammation.

In a new study, Manfred Thiel et al., in a team led by Michail Sitkovsky, test the hypothesis that oxygenation weakens a tissue-protecting mechanism triggered by hypoxia. Working with gene-altered mice, the team of immunologists, pathologists, and biochemists finds evidence that clinical oxygenation treatments could aggravate lung injury by inhibiting this protective pathway. But this protective pathway could potentially be restored, they argue, by artificially activating the inhibited pathway with therapeutic activators. Their results have important implications for how patients with ARDS and other serious lung diseases should be treated.[Fig pbio-0030211-g001]

**Figure pbio-0030211-g001:**
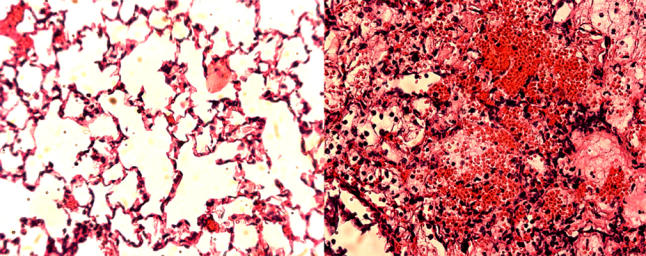
Lung tissue under ambient oxygen levels (left) and under 100% oxygen (right), which exacerbates acute inflammatory lung injury

Hypoxia triggers a signaling pathway mediated by an adenosine receptor (called A_2A_R) that arrests inflammation and tissue damage. It's thought that this same hypoxia-driven pathway protects the lungs from the toxic effects of overactive immune cells called neutrophils. Using a mouse model of acute lung injury induced by bacterial infection, Thiel et al. exposed one group of mice to 100% oxygen, mimicking therapeutic oxygenation, and left another group at normal ambient levels (21% oxygen). Five times more mice died after receiving 100% oxygen than died breathing normal oxygen levels. Mice given 60% oxygen—considered clinically safe—got worse, but didn't die.

To test the hypothesis that oxygen was precipitating these drastic results by exacerbating tissue inflammation, the authors analyzed the neutrophil-mediated immune response. Establishing a correlation between high neutrophil count and increased capillary leakage—indicated by the protein concentrations recovered from the alveolar space, which mediates gas exchange—Thiel et al. confirmed that overactive neutrophils promote lung injury. When otherwise healthy mice were subjected to lung infection and treated with hypoxia (10% oxygen), after 48 hours 90% of the mice showed several signs of improvement associated with an inhibition of neutrophil-mediated inflammation.

Thiel et al. went on to show that the adenosine receptor pathway was involved in the oxygen-dependent inflammation. By isolating neutrophils from mice with inflamed lungs and exposing them to high concentrations of a molecule that activates the adenosine receptor, they triggered increased levels of both A_2A_R and cAMP, a molecule that inhibits inflammation. No such increases were seen in mutant mice lacking functional A_2A_R proteins.

Hypoxia protects against lung damage, the authors conclude, by working through the A_2A_R signaling pathway to control inflammation. Above-normal oxygen levels interrupt this anti-inflammatory pathway, paving the way for further lung injury. Administering a molecule that jump-starts A_2A_R signaling artificially also significantly reduced the pathological side effects of oxygenation. These results may help explain why some patients with ARDS die following oxygenation therapy. And by identifying the mechanism that is disrupted by oxygenation—A_2A_R signaling—this study suggests that therapies aimed at activating the anti-inflammatory A_2A_R pathway may allow patients to receive the benefits of oxygenation therapy without succumbing to its toxic effects.

